# Marker-controlled watershed with deep edge emphasis and optimized H-minima transform for automatic segmentation of densely cultivated 3D cell nuclei

**DOI:** 10.1186/s12859-022-04827-3

**Published:** 2022-07-21

**Authors:** Tuomas Kaseva, Bahareh Omidali, Eero Hippeläinen, Teemu Mäkelä, Ulla Wilppu, Alexey Sofiev, Arto Merivaara, Marjo Yliperttula, Sauli Savolainen, Eero Salli

**Affiliations:** 1 HUS Medical Imaging Center, Radiology, Helsinki University Hospital and University of Helsinki, P.O. Box 340, FI-00290 Helsinki, Finland; 2 Department of Physics, University of Helsinki, P.O. Box 64, FI-00014 Helsinki, Finland; 3 HUS Medical Imaging Centre, Clinical Physiology and Nuclear Medicine, Helsinki University Hospital and University of Helsinki, Helsinki, Finland; 4 Division of Pharmaceutical Biosciences, Faculty of Pharmacy, Centre for Drug Research, University of Helsinki, Helsinki, Finland

**Keywords:** Nuclei, U-net, Watershed, H-minima

## Abstract

**Background:**

The segmentation of 3D cell nuclei is essential in many tasks, such as targeted molecular radiotherapies (MRT) for metastatic tumours, toxicity screening, and the observation of proliferating cells. In recent years, one popular method for automatic segmentation of nuclei has been deep learning enhanced marker-controlled watershed transform. In this method, convolutional neural networks (CNNs) have been used to create nuclei masks and markers, and the watershed algorithm for the instance segmentation. We studied whether this method could be improved for the segmentation of densely cultivated 3D nuclei via developing multiple system configurations in which we studied the effect of edge emphasizing CNNs, and optimized H-minima transform for mask and marker generation, respectively.

**Results:**

The dataset used for training and evaluation consisted of twelve in vitro cultivated densely packed 3D human carcinoma cell spheroids imaged using a confocal microscope. With this dataset, the evaluation was performed using a cross-validation scheme. In addition, four independent datasets were used for evaluation. The datasets were resampled near isotropic for our experiments. The baseline deep learning enhanced marker-controlled watershed obtained an average of 0.69 Panoptic Quality (PQ) and 0.66 Aggregated Jaccard Index (AJI) over the twelve spheroids. Using a system configuration, which was otherwise the same but used 3D-based edge emphasizing CNNs and optimized H-minima transform, the scores increased to 0.76 and 0.77, respectively. When using the independent datasets for evaluation, the best performing system configuration was shown to outperform or equal the baseline and a set of well-known cell segmentation approaches.

**Conclusions:**

The use of edge emphasizing U-Nets and optimized H-minima transform can improve the marker-controlled watershed transform for segmentation of densely cultivated 3D cell nuclei. A novel dataset of twelve spheroids was introduced to the public.

**Supplementary Information:**

The online version contains supplementary material available at 10.1186/s12859-022-04827-3.

## Background

Confocal fluorescent imaging has become a leading imaging method to study cell samples and imaging in 3D is essential to fully understand the cell morphology and changes in it. The segmentation of cells or nuclei is a critical requirement for quantitative analysis. Segmentation is a prerequisite for extracting morphological shape features like volume, surface area, elongation and roundness or sphericity of individual nuclei. While analyses in 2D have been the standard for a long time, in recent years the interest in 3D modelling has been growing rapidly. 3D cell models have several advantages over 2D cell cultures. In the 3D model, cells start to express their natural organisational structures and extracellular matrices [1]. The complex structure also leads to nutrient and oxygen heterogeneity within the model, which are found also in vivo. It has been shown that in 3D cultured cells are in a vivo-like state, which can be seen in their gene expression and cell behaviour. One of the most commonly used 3D culture system is the spheroid model, which is used in drug research [2, 3]. The blurred and sparse cell boundaries as well as high anisotropy of images are important challenges associated with the 3D segmentation.

Targeted molecular radiotherapies (MRT) allows targeting of high absorbed radiation doses selectively at a cell-level [4]. The importance of cell-level dosimetric modelling has been recognised already for a long time since the information about the dose-response relationships is needed to optimise MRT. The modelling has been shown to be viable with segmented cell nuclei [5]. If the segmentations of nuclei were available, nuclei staining could be utilised to differentiate living and dead cells, for example by Hoechst 33342 and propidium iodide dual-staining [6].

While many approaches have been proposed for cell segmentation [7–14], one of the most commonly used has been the marker-controlled watershed transform [15]. This watershed approach transforms the input, consisting of cell masks and markers, to an instance segmentation which assigns different labels for the voxels of separate instances of nuclei [16]. In the instance segmentation each distinct nuclei is detected and delineated. This is a more complicated task than semantic segmentation in which each voxel is classified to one of the pre-defined classes. Creating the binary cell masks is an example of semantic segmentation. Both the masks and markers are generated via an external algorithm. One of the challenges of this approach is the formulation of markers which, especially with densely clustered cells, is often difficult. To address this issue, the use of H-minima transform as a method for assigning markers from optimally suppressed regional minima of the input image, e.g. distance transformed cell masks, have been proposed [15, 17, 18].

In recent years, approaches exploiting deep learning in the form of convolutional neural networks (CNNs) have also been used to generate markers and cell masks with state-of-the-art results [19–28]. The most popular CNN architecture used in these approaches has been U-Net [29]. Deep learning based segmentation has been widely used in other fields, e.g. in medical imaging 3D segmentation is an extremely important task (for reviews, see e.g. [30, 31]). In the cell segmentation, especially the CNN-based generation of cell masks has been successful, addressing another problem of the marker-controlled watershed, a correct cell delineation. To enhance cell delineation, various approaches for creating edge emphasizing masks via CNNs have been proposed both in 2D [29, 32, 33] and 3D [34]. The aim of such masks is to separate clustered cells via edges. This, however, can be problematic, as a misdetection of single boundary pixel may lead to the fusion of touching nuclei. To alleviate this problem, various loss functions which emphasize cell boundaries [29, 32, 34, 35] and the use of edge emphasizing masks alongside watershed algorithm have been proposed [25, 34, 36]. However, modified loss functions and improved neural network architectures proposed in the literature almost always include tuning of a set of hyperparameters. On the other hand, H-minima transform has only one parameter and is therefore straightforward to optimize. Consequently, the idea of combining H-minima-based marker-controlled watershed and mask and marker generating CNNs is reasonable and interesting. However, to our knowledge, such combinations for 3D nuclei segmentation are yet to be investigated.

In this study, we devised several system configurations where marker-controlled watershed was combined with CNNs and optimized H-minima transform for 3D nuclei segmentation. Namely, we first constructed a baseline method where two 3D U-Nets [37] created binary nuclei mask and seeds. The seeds were transformed into markers via connected component analysis and, thereafter, markers and masks were fed to marker-controlled watershed [22, 25] which performed instance segmentation. The target seeds were eroded versions of nuclei masks. The system configurations were designed as modifications of this method, either replacing the nuclei masking U-Nets with edge emphasizing 2D or 3D U-Nets, connected component analysis with H-minima-based marker-controlled watershed or by excluding the creation of seeds and assigning markers by applying H-minima transform on nuclei mask. The aim of edge emphasizing U-Nets was to create binary nuclei mask in which borderlines of clustered cells were segmented as background. The depth parameter in the H-minima transform was optimized based on a novel metric describing an overall roundness of the instance segmentation.

The experiments were performed using densely packed twelve HepG2 nuclei spheroids for training and evaluation in a cross-validation scheme and also four other datasets for evaluation. All samples of the datasets were expanded near isotropic. In addition to the baseline, the system configurations were compared to other H-minima-based marker-controlled approaches where nuclei mask were generated with traditional methods and also to multiple well-known reference methods or software.

## Methods

### Pre-processing

First, the original slices are downsampled or upsampled, depending on the size of input slices, to the size of $256 \times 256$ pixels using bi-linear interpolation. If downsampled, the slices are lowpass filtered by the 2D Gaussian filter ($\sigma =1$ pixel) before downsampling to prevent aliasing and to decrease noise. Thereafter, slices containing no or only minimal signal are detected and zeroed to exclude background in model training. A slice is defined to belong to the background if the maximum intensity of the slice is less than $10\%$ of the maximum intensity of the whole image stack, and if it is additionally either the first or last slice in the stack or connected to another background slice. The detection of background is applied to the slices processed by the Gaussian filter ($\sigma =3$ pixels). The filtering is performed to decrease the effect of isolated high-intensity peaks in the background detection. The choice of $\sigma =3$ pixels was experimentally discovered to be large enough to attenuate isolated focal intensity peaks but small enough not to diminish small nuclei.

New slices between the original slices are added by interpolation to make the data more isotropic. The images before the interpolation of new slices define the *input space* and the interpolated *expanded image stacks* define the *expanded space*. The expansion is performed using odd integer factors, usually with a factor 3, instead of resampling the voxels precisely isotropic. In this way, the original slices are included in the expanded stack, which makes the transformation back to the *input space* straightforward. In the expansion, *expandImageFilter* of the Insight Toolkit with linear interpolation mode is used [38].

### Masking with U-nets

**Fig. 1:**
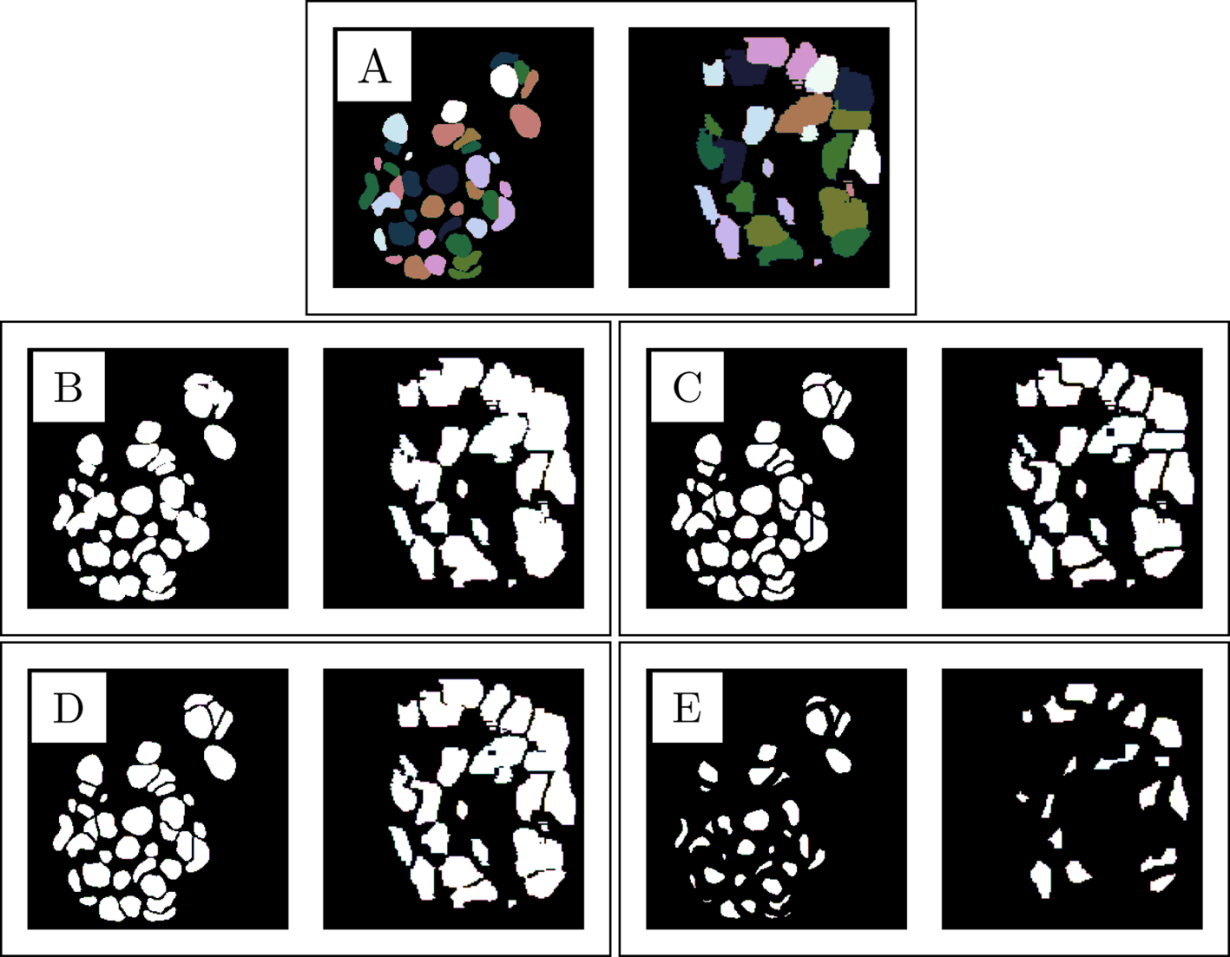
Visualization of an expanded ground truth, target nuclei masks and seeds in x-y- and x-z-planes. **A**: an expanded ground truth, **B**: 3D nuclei mask $M_{3D}$, **C**: 3D edge emphasizing nuclei mask $M_{3DE}$, **D**: 2D edge emphasizing nuclei mask $M_{2DE}$ and **E**: the seeds *S*. The actual height and width of each image are both 87 $\mu$m

**Fig. 2:**
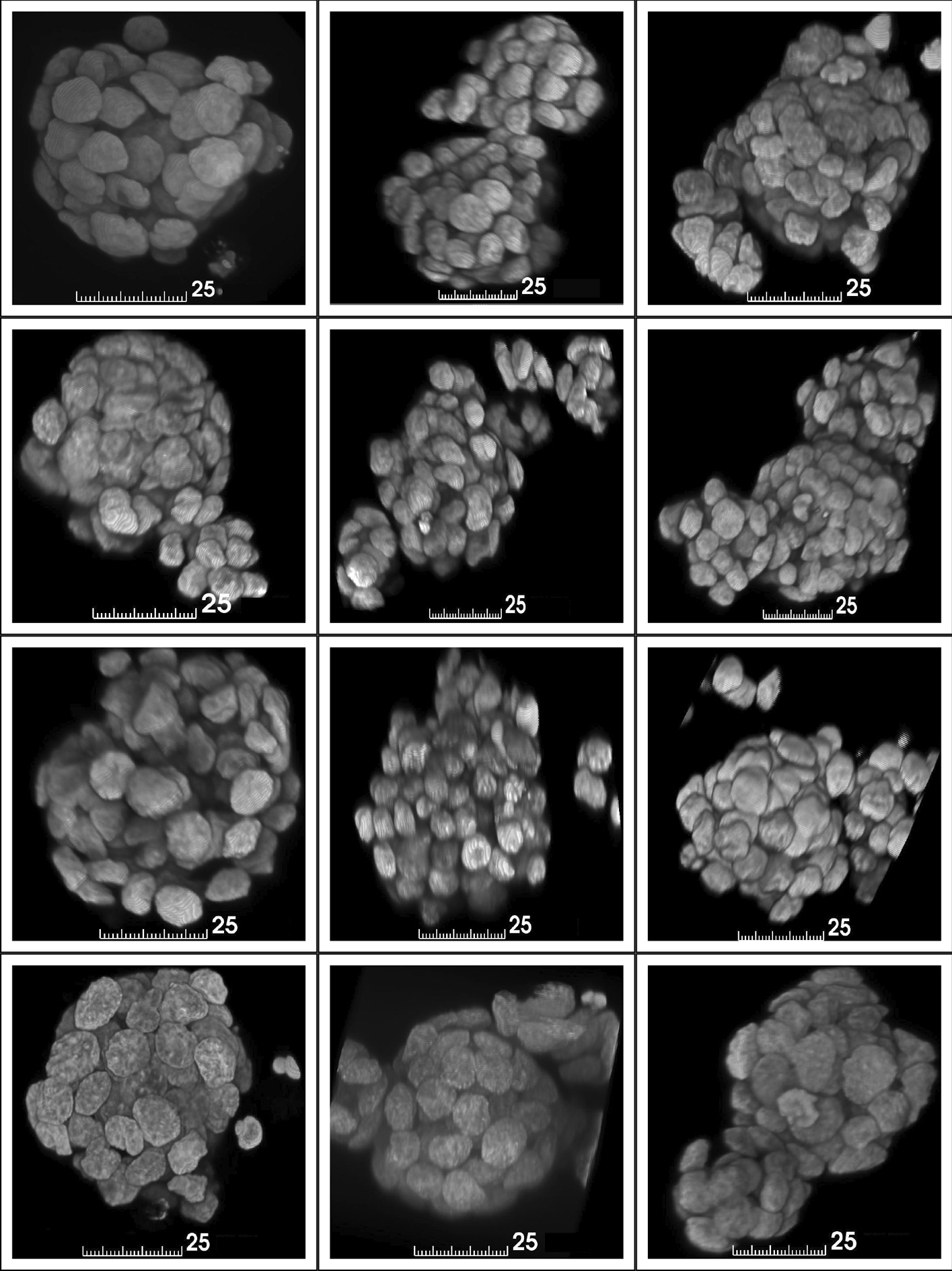
Volume renderings of the twelve 3D HepG2 nuclei spheroids. The unit for scale bars is $\mu$m

In this study, masking refers to an operation where the expanded input volume is transformed into binary nuclei masks or binary seeds *S*. Three different nuclei mask types are experimented: 3D nuclei mask $M_{3D}$, 3D edge emphasizing nuclei mask $M_{3DE}$ and 2D edge emphasizing nuclei mask $M_{2DE}$. Examples of the 3D predictions of these masks are illustrated in Fig. 1. The transformation is executed with either 2D or 3D U-Nets which were trained using target mask types and seeds. The U-Nets with target types consisting either of $M_{3DE}$ or $M_{2DE}$ are referred to as edge emphasizing U-Nets. Target mask types and seeds are visualized in Fig. 2 and their automatic generation from the manually defined ground truths is explained in Section *Target nuclei masks and seeds*.

The architecture of the 2D U-Nets is essentially the same as proposed by Ronneberger et al. [29] but the number of encoders is set to 5 and the number of decoders to 4. Moreover, the last layer of the network is a fully connected layer. The number of filters in *k*th encoder and decoder blocks were 28*k* and $28(5-k)$, respectively. Rectified linear unit activation is used after each convolutional layer, the filter size is $3\times 3$ and zero padding used to keep the sizes of the input and output of the layer the same. The input and output dimensions are $256 \times 256$.

The architecture of the 3D U-Nets follows the one in the 2D U-Nets, but 2D convolution layers are replaced with 3D convolution layers with $3\times 3\times 3$ filter size. The input and output dimensions are $256 \times 256 \times 24$. Upsampling and max-pooling are performed with $2 \times 2\times 1$ factors. To ensure that the number of parameters would be about the same between the 2D and 3D U-Nets, and thus make the comparison 2D versus 3D U-Net architecture more meaningful, the number of filters in *k*th encoder and decoder blocks are lowered to 16*k* and $16(5-k)$. The number of parameters is $3.3\times 10^6$ and $3.2\times 10^6$ in 2D and 3D U-Nets, respectively.

Using the two architectures, we formed four model types which each had a different target type: $U_{M_{3D}}$, $U_{M_{3DE}}$, $U_{S}$ and $U_{M_{2DE}}$, where the footnote refers to the target type. Each model performs masking patch-wise with the input image divided to the patches with the same dimensions as the input dimensions of the input of the model. If the size of the image is not divisible by the patch size, the last and the second last patch have overlap to ensure that the whole image is segmented. The prediction of $U_S$ is always multiplied with the prediction of $U_M$, where $M \in \{M_{3D}, M_{3DE}, M_{2DE}\}$ to ensure that the predicted seeds would not continue outside the predicted nuclei mask. The predictions of $U_M$ are thresholded with 0.5 and $U_S$ with 0.3. The threshold for seeds is lower due to the multiplication with nuclei mask and to alleviate the problem of vanishing of small sized seeds. The problem was encountered in our preliminary experiments with the twelve spheroids discussed in Section *Datasets*, especially when some of the nuclei were only partially visible in the image.

### Watershed methods

The components of the studied watershed methods are 3D morphological marker-controlled watershed ($WS_m$), H-minima-based marker-controlled watershed ($WS_h$) and 3D connected components filter (*CC*). $WS_m$ requires an input consisting of markers and nuclei mask and first applies Euclidean distance transform (DT) on the mask. The spacings of the transform (pixel dimensions) $\{s_{xy}, s_{xy}, s_z\}$ are normalised to $\{\frac{s_{xy}}{s_{xy}}, \frac{s_{xy}}{s_{xy}}, \frac{s_z}{s_{xy}}\}$. Then, DT and markers are fed to a morphological watershed transform which produces instance segmentation. $WS_h$ operates otherwise similarly but assigns markers from DT, constructed either from a nuclei mask or seeds, using H-minima transform with a given *h*-value. The transform suppresses “weak” local minima in DT with the depth parameter, *h*-value, reducing oversegmentation [15, 39]. *CC* transforms seeds into markers by assigning voxels of isolated seeds with a unique label.

The watershed methods, visualized as block diagrams in Fig. 1, are defined as

1 $$\begin{aligned}&{\textbf {A:}} O = WS_m(M, CC(S)), \end{aligned}$$

2 $$\begin{aligned}&{\textbf {B:}} O = WS_h(M, h), \end{aligned}$$

3 $$\begin{aligned}&{\textbf {C:}} O = WS_m(M, WS_h(S, h)), \end{aligned}$$

where *O* denotes instance segmentation. The method *A* is essentially $WS_m$, but markers are created from binary seeds with *CC*. The method *B* is the same as $WS_h$. The method C is similar to A but the markers are not assigned with a connected component analysis but by applying H-minima-based marker-controlled watershed to the seeds *S*. In essence, the method *C* uses watershed twice, first for seeds to form markers and then for these markers and a given mask. Instance segmentations are post-processed to exclude objects which are $5\%$ or less of the average volume of all segmented objects.

### Estimation of optimal H-value

All watershed methods utilizing H-minima transform are computed using *N* different values for parameter *h* producing a set of segmentation proposals $\{O_1, ..., O_N\}$. The set of *h*-values is fixed to $\{1.0, 1.25, 1.5, 1.75, 2.0, 2.5, 3.0,4.0,5.0\}$ ($N=9$). The minimum value of the range is set to 1.0 that corresponds to the size of one voxel in all data sets since the sizes of voxels were normalised. The choice of the maximum value is explained in Section *Experiments on the datasets*.

To choose the best segmentation, the roundness value *R* of each segmented object in the given segmentation $O_i$ is calculated. *R* is computed using the implementation of *LabelShapeStatisticsImageFilter* of the *SimpleITK*. Then, the average roundness score $\phi _R$ is calculated. The segmentation with the highest $\phi _R$ is chosen as the final segmentation. The intuition behind this score is to penalise segmentations that contain non-spherical segmented objects likely formed by many nuclei clumped together.

### Datasets

**Fig. 3:**
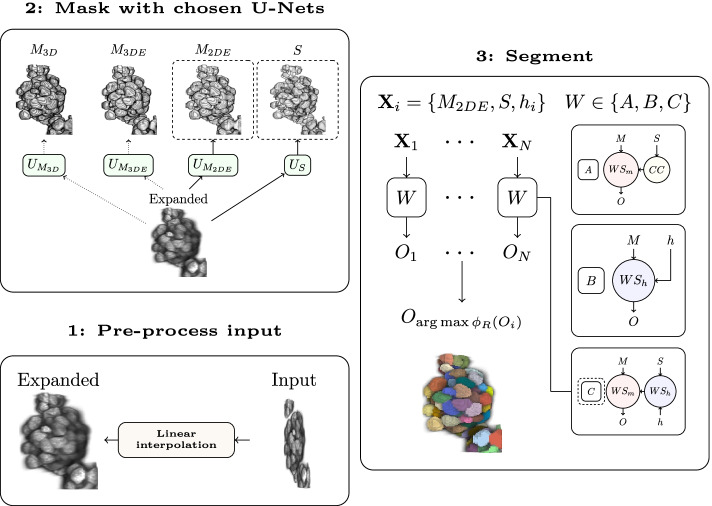
Segmentation of nuclei via system configurations with a demonstration using the configuration $\{C, M_{2DE}, S\}$. All the configurations utilise an input volume that is expanded near isotropic (1). The expanded volume is transformed with a chosen U-Net model type ($U_{M_{3D}}, U_{M_{3DE}}, U_{M_{2DE}}$ or $U_{S}$) into one of the nuclei masks $M \in \{M_{3D}, M_{3DE}, M_{2DE}\}$, where $M_{3D}$ denotes 3D nuclei mask, $M_{3DE}$ 3D edge emphasizing nuclei mask and $M_{2DE}$ 2D edge emphasizing nuclei mask, and optionally to binary seeds *S* (2). Instance segmentation is performed using one of the three different marker-controlled watershed methods *A*, *B* or *C* (3). The method *A* transforms binary seeds into markers via connected component (*CC*) analysis, and feeds markers and nuclei mask to the marker-controlled watershed transform, $WS_m$, which computes distance transform (DT) of nuclei mask and creates an instance segmentation. The method *B* uses H-minima-based marker-controlled watershed, $WS_h$, which input consist of nuclei mask and a *h*-value. Markers are determined from the nuclei mask via DT and H-minima transform, and similarly as in $WS_m$, DT and markers are transformed into an instance segmentation. The method *C* is otherwise the same as *A* but generates markers by feeding seeds to $WS_h$. Given a mask, optionally seeds and *N* different *h*-values, a chosen watershed method produces *N* different segmentation maps $O_i$. The segmentation $O_i$ with the highest average roundness score $\phi _R$ is chosen as the final segmentation (3)

The used datasets are referred as the twelve HepG2 spheroids and the independent datasets. The spheroids consist of twelve 3D HepG2 nuclei spheroids which were imaged with a confocal microscope and are visualized in Fig. 3. The spheroids are introduced to the public first time in this study. The x-y-plane resolutions varied from 0.07 to 0.1 $\mu$m and the step size for forming z-stack was 1.01 $\mu$m. The original size of the image slices was $1024 \times 1024$ pixels. More detailed descriptions of the imaging parameters and the cultivation of the spheroids are given in Additional file 1. The essential feature of this datasets was the clumped nature of the nuclei, making even manual segmentation challenging.

The independent datasets include Neurosphere [40, 41], Embryo [40, 41], monolayer of induced pluripotent human stem cells (hiPSC) BBBC034v1 Thirstrup et al. 2018, available from the Broad Bioimage Benchmark Collection [42] and a 3D HepG2 spheroid of liver cancer cells [5]. In our study, only the nuclear staining channel of each dataset was used. See the Additional file 1 for more details.

The ground truth segmentations of the twelve spheroids were defined manually using the Segment editor of 3D Slicer image computing platform [43]. The process is described with more details in Additional file 1. The ground truth segmentation for the liver data was performed by Reijonen et al. [5]. The ground truths of the other three independent datasets were downloaded from the public websites [40, 42].

### Target nuclei masks and seeds

Target nuclei masks and seeds for neural network training were created automatically using the expanded or original ground truths of the twelve spheroids. The ground truths were transformed from *input space* to *expanded space* via morphological contour interpolation method [44]. The idea of the method is to give a smooth change of shape between the slices. However, we found that the nuclei that were originally connected in z-direction were not necessarily connected after interpolation. In the between-two nuclei regions, defined using a morphological closing operation, we modified the original contour interpolation method to use nearest-neighbourhood interpolation to avoid extra gaps between nuclei.

Nuclei masks of type $M_{3D}$, $M_{3DE}$ and seeds *S* were generated from the expanded ground truths. $M_{3D}$ masks were simply binarized versions of the expanded ground truths. The edge emphasized versions, $M_{3DE}$ masks, were otherwise the same but also the outer boundaries, i.e. edges, of each nucleus were set to value 0 to separate touching nuclei by a background stripe. The edges were computed using *find_boundaries* (mode *outer*) method in *scikit-learn* [45].

To create the seeds *S* for a given spheroid, a morphological binary erosion operation (spherical structuring element with radius $r=3$ voxels) was applied to each nucleus of the expanded ground truth of the spheroid separately. If the erosion split an original nucleus into two or more parts, a lighter erosion ($r=1$ voxel) was used instead. In the rare cases when lighter erosion also split the nucleus, erosion was performed only on x-y plane (spherical structuring element $r=1$ pixel). Morphological erosion was used because it approximately retains the shape of the nuclei in the seeds. The seeds *S* were then computed as a binarized version of the eroded image.

Nuclei masks of type $M_{2DE}$ were computed from original ground truths by first binarizing them and then by using the 2D version of *find_boundaries*. The method was applied to each xy-slice of given binarized ground truth and it created 2D-based edges which were labeled as background. Different types of target masks and seeds are illustrated in Fig. 2.

### Training of U-nets

A total of 12 models of each U-Net model type discussed in Section *Masking with U-Nets* were trained using the twelve expanded spheroids and the corresponding binary targets. In essence, a given model type $U_T$ where $T \in \{M_{3D}, M_{3DE}, M_{2DE}, S\}$ had twelve different models $U_T^m$, where $m \in \{1,...,12\}$. When $m=1$, the validation set of a model was the 12th spheroid and (*m*-1)th spheroid otherwise. The *m*th spheroid was always left out of the training and used for evaluation as discussed in Section *System configurations*. The other 10 spheroids were used for training.

In the training of a given $U_T^m$ with 3D U-Net architecture, training samples consisted of input, $256\times 256\times 24$ sized expanded spheroid patch and target *T* patch with the same dimensions. Input patches were extracted from the expanded spheroids using a sliding window with 12 slices overlap. When the sliding patch window partly crossed the image volume boundaries, zeros were added to fill the patch. Target patches were extracted similarly from the expanded ground truth. The intensities of the input patch were normalised between [0, 1]. Keras framework [46] (version 2.3.0) was used for training the models. The number of epochs was 200, batch size 4, the loss function binary cross entropy, and the optimizer Adam [47]. The loss function and optimizer were initialized with default parameters of their Keras implementations. The initial learning rate was 0.001 which was decayed by factors of five and ten after 75 and 110 epochs, respectively. Augmentation was performed on-the-fly during training: axial rotations with 360 degrees range were performed to $67\%$ of samples in a training set batch and mirroring to $50\%$ of samples in the batch. The final model configuration was chosen based on the epoch with the lowest validation loss. This training process was replicated for each 3D-based U-Net type discussed in Section *Masking with U-Nets*.

The dimensions of target and input patches, or slices, were $256\times 256$ when training $U_{M_{2DE}}^m$ models. The input slices and targets were extracted from the original spheroids and ground truths. The batch size was set to 16. With this choice, the number of updates was essentially the same as when training 3D U-Net models. Otherwise, the training configuration was the same as explained in the previous paragraph. Using Tesla V100 GPU, the training time for an epoch of 3D U-Net type model was about 50 seconds and 10 seconds for 2D U-Net type model.

### System configurations

The system configurations are denoted either with $\{w, M, s\}$ or with $\{w, M, s, *\}$, where $w \in \{A, B , C\}$, $M \in \{M_{3D},M_{3DE}, M_{2DE} \}$ and $s \in \{-,S\}$. The configurations $\{w, M, s\}$ were evaluated on the twelve spheroids: keeping the *w*, *M* and *s* fixed, the masks of each *m*th spheroid were created with $U_M^m$ and, depending on the configuration, with $U_S^m$. The *m*th spheroid was always left out of the training of each $U_M^m$ and $U_S^m$ model. The evaluation set of the configurations $\{w, M, s, *\}$ consisted of the independent datasets. These configurations are otherwise the same as $\{w, M, s\}$ but have twelve different settings with $m \in \{1, ..., 12 \}$.

### Deep learning baselines

The configuration $\{A,M_{3D},S\}$ is named as U-Nets+SWS and $\{A,M_{3D},S,*\}$ as U-Nets+SWS*. Here, we inherit the notation from [25], with SWS refering to seeded i.e. marker-controlled watershed. In this study, they can be considered as baseline methods previously introduced in the literature and similar to QCANet [22].

Additionally, we devised 3D versions of U-Net proposed in [29]. These versions, named as U-Net-Cell and U-Net-Cell* similarly as in [35], were similar to $\{B,M_{3DE},-\}$ and $\{B,M_{3DE},-, *\}$ but with two main differences: instance segmentations were produced using connected component analysis and the loss function used in training of the edge masks was a 3D version of weighted cross entropy loss introduced in [29]. In this loss function, voxels representing boundaries of clumped nuclei were given higher weight. The parameters of the loss function were otherwise the same as in [29] but the weights of nuclei and background classes were 3 and 1 to address class imbalance, respectively.

### Evaluation metrics

The evaluation metrics used in this study are Panoptic Quality (PQ) [48], Jaccard Index (JI), Aggregated Jaccard Index (AJI) [49] and nuclei number difference percentage (NNDP). The first three are based on an intersection over union (IoU) which is defined as $\text {IoU}(X,Y)=|(X \cap Y )|/ |(X\cup Y)|$ where *X* and *Y* are the set of voxels belonging to the nuclei, in the segmentation and ground truth. The nuclei are considered matching nuclei if $\text {IoU}(X,Y)>0.5$. The detection quality (DQ) measure is the object-level F1 score: $\text {F1}=\frac{TP}{TP+0.5(FP + FN)}$ where TP is the number of nuclei in the segmentation that have match in the ground truth, FP is the number of nuclei in the segmentation that do not have match and FN is the number of nuclei in the ground truth that do not have match in the segmentation. Segmentation quality (SQ) measures how accurately the nuclei are delineated and it is defined as the average IoU of matched nuclei. PQ is defined as product of F1 Detection Quality (DQ) and Segmentation Quality (SQ): $\text {PQ}=\text {DQ} \times \text {SQ}$. We used PQ as main evaluation metric because it measures both the detection quality and segmentation accuracy in one score. Additionally, [50] presented an opinion that PQ should be a standard performance measure for nuclear instance segmentation methods.

Jaccard index, like SQ, is defined as the average of IoUs of matched objects. However, in the implementation provided by Piccinini et al. [40] the IoU was calculated for every nucleus of the ground truth and its matched nuclei was the one that maximized IoU. We use this formulation of JI to enable direct comparison with the results in [40]. The drawback of JI is that false positive nuclei detections are not penalised. Aggregated Jaccard index (AJI) [49] was originally designed to be an enhanced version of JI, and it uses the voxel count of false positive nuclei to penalise its value. AJI is used as an evaluation metric in many nuclei instance segmentation studies and thus helpful to compare our results with the literature.

In addition to PQ, JI and AJI, we defined nuclei number difference percentage (NNDP) as

4 $$\begin{aligned} \text {NNDP} = \frac{2|S_{\text {num}} - G_{\text {num}}|}{S_{\text {num}} + G_{\text {num}}} \times 100\%, \end{aligned}$$

where $S_{\text {num}}$ is the number of cell nuclei in the segmentation and $G_{\text {num}}$ in the ground truth.

### Experiments on the datasets

The segmentations obtained using the configurations discussed in Section *System configurations* were compared to the ground truths using the defined evaluation metrics. With the twelve spheroids we used all metrics whereas with the independent datasets we computed PQ and JI. Before computing the evaluation metrics, the segmentations were always transformed from *expanded space* to the size of the original ground truths. This was performed by choosing matching slices and via 2D nearest-neighbourhood interpolation or extrapolation on the x-y-plane when the dimensions of this plane differed from $256\times 256$. All the experiments were performed using twelve CPUs (Intel Xeon Gold 6248) without GPU support. Using the computationally heavy configuration $\{C,M_{3DE},S,*\}$, the creation of mask and seeds, instance segmentation and computation of evaluation metrics for the 5th spheroid consisting of 141 nuclei took about three minutes.

Using the twelve spheroids, we made an experiment where the roundness score $\phi _R$ was replaced with $\frac{\text {AJI}+\text {PQ}+\text {JI}}{3}$ in the estimation of the *h*-value. In preliminary experiments, the score, named as the optimal score, was used to determine the highest *h*-value of the estimation range discussed in Section *Estimation of optimal h-value* using the $\{B, M_{3DE}, -\}$, $\{C, M_{3DE}, S\}$ and $\{C, M_{3DE}, S\}$ and the twelve spheroids. In the end, this highest optimal *h*-value was found to be 5.0 with the eleventh spheroid.

In addition to the deep learning baselines, we constructed four different H-minima transform-based marker-controlled watershed approaches for results comparisons: WS, aifWS, nlWS and blWS. The baselines were used to segment the twelve spheroids. All the baselines used the watershed method B discussed in Section *Watershed methods* and performed masking by Otsu’s thresholding method [51]. aifWS, blWS and nlWS used ITK [38] implementations of various non-linear denoising techniques: gradient anisotropic diffusion filtering (aifWS) [52], bilateral filtering (blWS) [53], and patch-based denoising (nlWS) [54]. Moreover, 3D CellProfiler pipeline was used as one of the baselines. The pipeline was tested both using the expanded data (CeP) and the original data as input (CeP non-exp). Each baseline had twelve parameter settings, one for each spheroid. Each of these settings was optimized to give the highest possible PQ score on a unique spheroid. More details of the optimization process are given in Additional file 1.

While we tested various system configurations on all datasets in the independent datasets, we used the Embryo and Neurosphere datasets to perform results comparisons with the software reported by Piccinini et al. [40]. Most of the algorithms were automatic but operated with human interaction, e.g. required tuning of some parameters. The best performing software in the paper was semi-automatic 3D-Cell-Annotator [41].

## Results

Table 1 presents the results of our experiments on the twelve spheroids using six different traditional algorithms, deep learning baselines and eight system configurations. CeP, using expanded data, was the best performing non-deep learning-based algorithm in all metrics. It was outperformed by all system configurations, U-Net-Cell and U-Nets+SWS which achieved over $20\%$ relative improvement on all average scores except the NNDP.

**Table 1:** The average evaluation scores and their standard deviations over twelve spheroids

Method	AJI	PQ	JI	NNDP ($\%$)
WS	0.47 ± 0.06	0.43 ± 0.06	0.53 ± 0.05	19.9 ± 10.9
aifWS	0.52 ± 0.07	0.50 ± 0.08	0.57 ± 0.05	16.3 ± 7.6
nlWS	0.47 ± 0.06	0.45 ± 0.05	0.53 ± 0.05	20.2 ± 10.1
blWS	0.50 ± 0.06	0.48 ± 0.07	0.56 ± 0.05	17.5 ± 8.6
CeP non-exp	0.34 ± 0.05	0.26 ± 0.06	0.41 ± 0.05	31.5 ± 19.8
CeP	0.53 ± 0.05	0.48 ± 0.06	0.58 ± 0.05	6.3 ± 5.5
$\text {U-Net-Cell}$	0.54 ± 0.15	0.57 ± 0.1	0.63 ± 0.09	16.6 ± 14.1
$\text {U-Nets+SWS}$	0.66 ± 0.09	0.69 ± 0.07	0.73 ± 0.06	11.4 ± 6.2
$A,M_{3DE},S$	0.72 ± 0.08	0.73 ± 0.07	0.76 ± 0.05	6.1 ± 4.6
$A,M_{2DE},S$	0.68 ± 0.08	0.71 ± 0.06	0.74 ± 0.05	9.9 ± 5.3
$B,M_{3D},-$	0.63 ± 0.1	0.65 ± 0.09	0.69 ± 0.08	14.6 ± 8.1
	(0.64 ± 0.11)	(0.66 ± 0.09)	(0.7 ± 0.08)	(12.6 ± 9.4)
$B,M_{3DE},-$	0.76 ± 0.06	0.75 ± 0.06	**0.78** ± 0.04	3.3 ± 2.2
	(0.77 ± 0.06)	(0.76 ± 0.06)	(0.78 ± 0.04)	(2.4 ± 1.2)
$B,M_{2DE},-$	0.7 ± 0.07	0.7 ± 0.07	0.74 ± 0.05	5.2 ± 3.1
	(0.71 ± 0.07)	(0.7 ± 0.07)	(0.75 ± 0.05)	(4.7 ± 3.6)
$C,M_{3D},S$	0.73 ± 0.09	0.74 ± 0.07	0.76 ± 0.06	5.6 ± 6.4
	(0.76 ± 0.06)	(0.75 ± 0.05)	(0.78 ± 0.04)	(2.7 ± 2.3)
$C,M_{3DE},S$	**0.77** ± 0.06	**0.76** ± 0.05	**0.78** ± 0.04	**1.8** ± 1.8
	(0.78 ± 0.06)	(0.77 ± 0.05)	(0.79 ± 0.04)	(2.2 ± 1.2)
$C,M_{2DE},S$	0.76 ± 0.05	**0.76** ± 0.05	**0.78** ± 0.04	2.4 ± 1.4
	(0.77 ± 0.06)	(0.76 ± 0.05)	(0.79 ± 0.04)	(1.8 ± 0.9)

The configurations are specified by $\{w,M,S\}$: *w* denotes watershed method, $M \in \{M_{3D},M_{3DE},M_{2DE}\}$, which denotes 3D mask, 3D and 2D edge emphasizing mask, respectively, and $S \in \{S,-\}$, which refers to use or exclusion of seeds. The scores in (.) brackets were obtained by replacing the maximization of roundness score $\phi _R$ with maximization of $\frac{\text {AJI}+\text {PQ}+\text {JI}}{3}$ in the determination of the *h*-value. These scores represent theoretically best scores obtained in an unrealistic scenario. AJI = Aggregated Jaccard Index, PQ = Panoptic Quality, JI = Jaccard Index, NNDP = nuclei number difference percentage. The best value for each evaluation metric is bolded

**Fig. 4:**
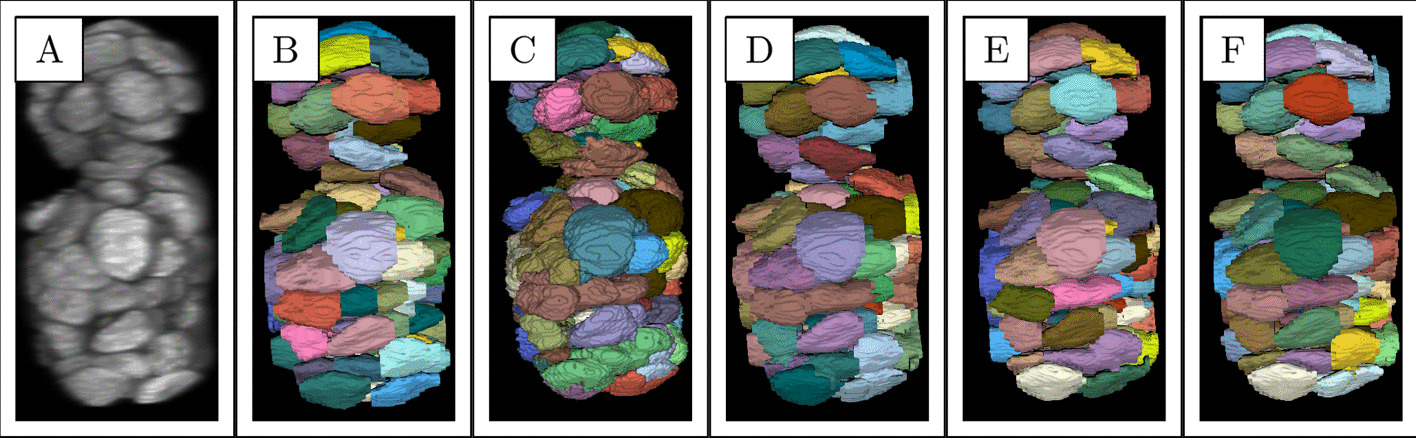
Results comparison on the 12th spheroid. **A**: the spheroid, **B**: ground truth, **C**: CellProfiler segmentation (PQ=0.51), **D**: U-Net+SWS segmentation (PQ=0.57), **E**: $\{C,M_{2DE},S\}$ segmentation (PQ=0.72), **F**: $\{C,M_{3DE},S\}$ segmentation (PQ=0.72. The configurations are specified by $\{w,M,S\}$: *w* denotes watershed method, $M \in \{M_{3D},M_{3DE},M_{2DE}\}$, which denotes 3D mask, 3D and 2D edge emphasizing mask, respectively, and $S \in \{S,-\}$, which refers to use or exclusion of seeds. Colors are arbitrary. The actual height and width of the spheroid are 86 $\mu$m and 39 $\mu$m, respectively. PQ = Panoptic Quality

Utilisation of the edge emphasizing U-Net models, especially the 3D ones, improved the results with all system configurations. The importance of the use of H-minima transform was slightly higher since the scores of $\{C,M_{3D},S\}$ were better than the scores of $\{A,M_{3DE},S\}$ or $\{A,M_{2DE},S\}$. When comparing U-Nets+SWS and $\{C,M_{3DE},S\}$ the relative improvement of the latter in terms of average $\frac{\text {AJI}+\text {PQ}+\text {JI}}{3}$ was $11\%$ and the standard deviation was smaller with all metrics. With $\{C,M_{3DE},S\}$ the NNDP was less than $15\%$ of the NNDP of $\{A,M_{3DE},S\}$. While $\{C,M_{3DE},S\}$ performed the best, $\{B,M_{3DE},-\}$ which did not utilise deep seeds and $\{C,M_{2DE},S\}$ which generated 2D edge emphasizing masks reached similarly high scores. All system configurations outperformed U-Net-Cell. Using the 12th spheroid, we provide visualized results comparison between CeP,U-Net+SWS and two highest performing configurations in Fig. 4.

The performance of the estimation of *h*-value was quantified by comparing to an approach where the roundness score $\phi _R$ was replaced with the optimal score $\frac{\text {AJI}+\text {PQ}+\text {JI}}{3}$. The results obtained with this score are presented in (.) brackets. The worst estimation was produced with $\{C,M_{3D},S\}$ in which mean NNDP increased from 2.7 to 5.6 and mean AJI decreased from 0.76 to 0.73 when using $\phi _R$ instead of the optimal score. In general, the use of the optimal score increased PQ, AJI and JI by approximately 0.01 units.

In Table 2, we illustrate the results on the independent datasets obtained by U-Net+SWS* and the three system configurations that achieved the three best scores on the twelve spheroids. Each configuration and deep learning baselines had twelve different settings: the average scores and their standard deviations obtained with the settings are reported. Using the Neurosphere and the Embryo datasets, we also provide a results comparison with the cell segmentation algorithms discussed an results reported in [40]. The system configurations improved the scores of U-Net+SWS* when evaluated on Neurosphere and Embryo but not with Liver and hiPSC. They outperformed U-Net-Cell* with all datasets. The best overall performing configuration was $\{C,M_{2DE}, S,*\}$ which in average beat or equaled the scores of the best performing algorithm, 3D-Cell-Annotator.

**Table 2:** PQ and JI scores on the independent datasets

Method	Neurosphere		Embryo		Liver	hiPSC
	PQ	JI	PQ	JI	PQ	PQ
IF3DImageJSuite [40, 55]	0.02	0.23	0.64	0.65	NA	NA
LoS [40, 56]	0.20	0.40	0.40	0.51	NA	NA
MINS [40, 57]	0.51	0.56	0.79	0.79	NA	NA
OpenSegSPIM [40, 58]	0.58	0.61	0.36	0.48	NA	NA
RACE [40, 59]	0.03	0.39	0.00	0.15	NA	NA
SAMA [40, 60]	0.00	0.12	0.40	0.49	NA	NA
Vaa3D [40, 61]	0.45	0.60	0.40	0.52	NA	NA
XPIWIT [40, 62]	0.59	0.62	0.73	0.74	NA	NA
3D-Cell-Annotator [40, 41]	0.64	0.69	**0.80**	0.80	NA	NA
$\text {U-Net-Cell}*$	0.51 ± 0.03	0.58 ± 0.02	0.64 ± 0.03	0.68 ± 0.02	0.67 ± 0.02	0.59 ± 0.04
$\text {U-Nets+SWS}*$	0.65 ± 0.01	0.68 ± 0.01	0.76 ± 0.03	0.77 ± 0.02	**0.71** ± 0.01	**0.75** ± 0.04
$B,M_{3DE},-,*$	0.66 ± 0.01	**0.70** ± 0.01	0.78 ± 0.01	0.78 ± 0.01	**0.71** ± 0.01	0.70 ± 0.16
$C,M_{3DE},S,*$	**0.67** ± 0.01	**0.70** ± 0.01	0.78 ± 0.01	0.78 ± 0.01	**0.71** ± 0.01	0.69 ± 0.14
$C,M_{2DE}, S,*$	0.66 ± 0.01	0.69 ± 0.01	**0.80** ± 0.02	**0.81** ± 0.02	**0.71** ± 0.01	0.74 ± 0.03

The configurations are specified by $\{w,M,S,*\}$: *w* denotes watershed method, $M \in \{M_{3D},M_{3DE},M_{2DE}\}$, which denotes 3D mask, 3D and 2D edge emphasizing mask, respectively, and $S \in \{S,-\}$, which refers to use or exclusion of seeds. We also report PQ and JI values of the reference software computed from the ground truth and result files provided by Piccinini et al. [40] for the Neurosphere and Embryo datasets. The configurations, U-Net+SWS* and U-Net-Cell* both had twelve different settings: the average scores and their standard deviations of these settings are illustrated. PQ = Panoptic Quality, JI = Jaccard Index. The best value for each dataset and evaluation metric combination is bolded

## Discussion

In this study, we implemented several novel system configurations which utilized U-Nets, optimized H-minima transform and marker-controlled watershed for 3D nuclei segmentation. The basis of the configurations was the the deep learning enhanced marker-controlled watershed method (U-Nets+SWS), similar to QCANet by Tokuoka et al. [22]. In this method, U-Nets were used to create nuclei masks and markers for the watershed algorithm. One difference between our baseline implementation and the one used in QCANet is that we used markers following nuclei shapes whereas QCANet used spherical markers. This enabled us to improve marker detection by applying watershed transform to the seed masks generated by U-Nets. By using various system configurations, we examined whether nuclei masks should be generated via edge emphasizing U-Nets, could the markers be defined from the nuclei masks using optimized H-minima transform or from binary seeds created by U-Nets using H-minima-based marker-controlled watershed. The depth parameter of the H-minima transform was optimized using overall roundness of a given segmentation.

All U-Nets were trained using standard architectures and a loss function which thus were not factors in the performance difference of the system configurations. Consequently, all results improvements were either due to the use of deep edge emphasis, optimized H-minima transform or both. While these methods have been discussed previously in the literature [15, 25, 33, 34, 36], their combination has not yet been tested. Experimenting with the combination is relevant since deep edge emphasis can be applied on top of any deep learning-based approach for masking and optimized H-minima transform in any postprocessing step which involves watershed algorithm.

In our experimental setup, all nuclei samples were expanded near isotropic using linear interpolation. The motivation for the expansion rose from our preliminary experiments in which we observed that the performance of the watershed algorithm was greatly improved when the input image was transformed isotropic. A similar situation occurred also with 3D CellProfiler as depicted in Table 1. Morphological contour-based interpolation was used to convert ground truth segmentations to near isotropic for the training of CNNs. These choices, along with the formulation of the shape and size of target seeds, enhanced the performance of the deep learning baselines and made them more robust. However, for this study, we opted to exclude reporting the preliminary experiments and focused on comparing various approaches and our system configurations in this fixed experimental setup.

The system configurations were tested on the dataset of twelve spheroids and the independent datasets. The results on the twelve spheroids in Table 1 demonstrate that the performance of the baseline method U-Nets+SWS was improved if U-Nets for nuclei masking were replaced by edge emphasizing U-Nets ($\{A,M_{3DE},S\}$ or $\{A,M_{2DE},S\}$). As the NNDP decreased and PQ increased with these choices, the edge emphasizing U-Nets contributed for both better marker assignments and nuclei delineation. In addition, replacing the connected component analysis of the baseline with the H-minima-based marker-controlled watershed had the same effects ($\{C,M_{3D},S\}$). The best results were achieved by combining both replacements ($\{C,M_{3DE},S\}$ or $\{C,M_{2DE},S\}$) with PQ increasing from 0.69 to 0.76 and NNDP decreasing from 11.4 to 1.8. The configuration in which markers were created from 3D edge emphasizing U-Nets with the help of H-minima transform (($\{B,M_{3DE},-\}$) reached almost the same scores as the two best systems watershed *C* method-based systems. Consequently, the creation of seeds was not a necessity.

U-Net+SWS, U-Net-Cell and all system configurations outperformed the traditional baselines. All traditional baselines, except CeP-based systems, used watershed method B but performed masking via filtering and Otsu’s thresholding. The parameters of these baselines were chosen optimally in an unrealistic setting. However, any system configuration using watershed method B significantly outperformed them. This result highlighted the importance of the generation of nuclei masks using U-Nets.

To the best of our knowledge, we are the first to compare the performance of 2D U-Nets to 3D U-Nets in a 3D nuclei segmentation task. The configuration $\{B,M_{2DE},-\}$ utilized only 2D edge emphasizing U-Nets and was shown to outperform the scores of U-Nets+SWS which used 3D U-Nets. However, if the U-Nets of this configuration were replaced with 3D edge emphasizing U-Nets, the results improved. On the other hand, 2D edge emphasizing U-Nets were as effective as their 3D counterparts with the configurations using watershed method *C*. Consequently, the results implied that relying solely on 2D U-Nets instead of 3D U-Nets would not be advisable, but that the former could be used for nuclei masking if markers were created from seeds via H-minima transform-based watershed.

The scores in () brackets in Table 1 illustrate the values that could have been attained if the *h*-value would have been chosen to maximise score $\frac{AJI+PQ+JI}{3}$ instead of the average roundness $\phi _R$ metric. In general, the use of average roundness to spsecify *h*-value was almost as effective. Even if more sophisticated methods for finding the best *h*-value, locally or globally, do exist [15, 17, 18], the use of $\phi _R$ metric was simple and demonstrated the benefit of the usage of H-minima transform in our system configurations.

The three best system configurations, based on the cross-validation using the twelve spheroids, as well as the deep learning baselines, were also evaluated using the independent datasets. The nuclei in these datasets were not highly clumped but their imaging protocols and nuclei types, excluding the Liver dataset, differed from the twelve spheroids. For this reason, the main purpose of the evaluation on the datasets was to test the ability of system configurations to generalize to data dissimilar to the training data. Each system configuration and the baseline U-Net+SWS* included twelve different U-Net settings. We reported the average scores and their standard deviations of these settings in Table 2. Using all settings was necessary to quantify the variation inherent in different settings trained with slightly different sets. The configurations outperformed U-Net-Cell* with all datasets and improved the scores of U-Nets+SWS* on Embryo and Neurosphere but not on Liver and hiPSC. Embryo and Neurosphere were previously investigated by Piccinini et al. [40] in which multiple well-known segmentation software were evaluated. The best performing software was 3D-Cell-Annotator [41] which scores we were able, in average, to outperform or equal using the configuration $\{C,M_{2DE},S,*\}$. This outcome is meaningful since 3D-Cell-Annotator was semi-automatic and required human interaction whereas the configuration operated fully automatically and was trained with dissimilar data. The edge emphasizing U-Nets, especially the 3D ones, were shown to fail to some extent with hiPSC. In this dataset the nuclei were not clustered and the U-Nets mistakenly assigned edges to individual nuclei and wrongly separated them. An obvious reason for this result is the disrepancy between the test and training sets.

Another highlight of this study was the comparison of 3D version of U-Net-Cell [29, 35] against $\{B,M_{3DE},-\}$ configuration. The configuration was otherwise the same as U-Net-Cell but did not utilize weight maps during training and produced instance segmentation using H-minima transform-based watershed algorithm. The configuration greatly outperformed U-Net-Cell on twelve spheroids as the average AJI improved from 0.54 to 0.76. Similar results were achieved over independent datasets with average PQ improving from 0.61 to 0.71. In a previous study with 3D nuclei dataset, U-Net-Cell was compared to an approach similar to U-Net-Cell but which used an enhanced loss function during training [35]. The use of the enhanced loss function led only to modest improvements as average AJI score improved from 0.63 to 0.66. In this perspective, our focus on using optimized H-minima transform to improve watershed algorithm instead of trying to enhance edge emphasizing U-Nets with different architecture or loss function solutions seemed justified.

A limitation of machine learning based methods is the uncertainty of generalizability to other types of data sets or to datasets acquired at other sites. In our tests, the independent datasets included three datasets in which the imaging protocols and the cell cultures were different than in our training sets. We achieved satisfactory results on these datasets. However, only datasets having visually similar image contrast and image features were chosen for this study. It is likely that if applied to datasets that differ substantially from the data used in training, the models will fail. Another limitation is the tedious work to create manually segmented ground truths, especially in the case of 3D volumetric data. The use of synthetic data [24] and sparse or weak annotations [37, 63] are examples of research directions that may alleviate the burden. However, manually annotated data is required for the quantitative evaluation of the methods.

## Conclusions

Our study provided evidence that marker-controlled watershed for 3D nuclei segmentation could be improved via using edge emphasizing U-Nets for mask and optimized H-minima transform for marker generation. The methods of deep edge emphasis and the use of H-minima transform are not tied to any specific neural network architecture or training procedure and can be exploited alongside virtually any deep learning-based nuclei segmentation approach in the future. The software and thirteen cell nuclei spheroids with manually segmented ground truths were made publicly available. Interesting future work directions could be the estimation of *h*-value locally instead of globally and the replacement of the roundness score with some other metric or a set of metrics. The training and evaluation sets should be enlarged to ensure the functionality of the system configurations with more diverse imaging protocols and cell cultures.

## Additional file

**Additional file 1:** Detailed information on the datasets used in the study is given and optimization process of the baseline algorithms is described.

## Data Availability

Software: https://doi.org/10.6084/m9.figshare.16595063 Prebuilt models: https://doi.org/10.6084/m9.figshare.16439157 Twelve spheroids: https://doi.org/10.6084/m9.figshare.16438314 Liver spheroid: https://doi.org/10.6084/m9.figshare.16438185.

## Abbreviations

2D: Two dimensional
3D: Three dimensional
AJI: Aggregated Jaccard Index
CNN: convolutional neural network
JI: Jaccard Index
NNDP: nuclei number difference percentage
PQ: Panoptic Quality
