# Supplementary material for: Marker-controlled watershed with deep edge emphasis and optimized H-minima transform for automatic segmentation of densely cultivated 3D cell nuclei
Source: BMC Bioinformatics. 2022 Jul 21;23:289. doi: 10.1186/s12859-022-04827-3 (PMC9306214; doi:10.1186/s12859-022-04827-3)
Supplement: Supplementary file 1 — Additional file 1: Detailed information on the datasets used in the study is given and optimization process of the baseline algorithms is described. [file 12859_2022_4827_MOESM1_ESM.pdf]

Supplementary materials for Marker-controlled Watershed with Deep Edge Emphasis and Optimized H-minima Transform for Automatic Segmentation of Densely Cultivated 3D Cell Nuclei

T. Kaseva B. Omidali E Hippeläinen T. Mäkelä
U. Wilppu A. Sofiev A. Merivaara M. Yliperttula
S. Savolainen E. Salli

1 Introduction

This document provides more detailed information on the datasets used in the study and describes the optimization process of the baseline algorithms.

2 Datasets

2.1 Cultivation and imaging of the twelve HepG2 spheroids

Human hepatocellular carcinoma HepG2 (HB-8065) cells were obtained from the American Type Culture Collection (ATCC, Manassas, Virginia, USA). The cells were maintained in 75 cm2 culture flasks in DMEM (31966-021, Gibco) supplemented with 10% fetal bovine serum, and pen-strep consisting of 100 IU/ml penicillin, and 100 μg/ml streptomycin. During the maintenance procedure, the cells were subcultured two times a week with the proportion of 1 to 5. HepG2 cell spheroids were cultured in nanofibrillated cellulose (NFC) hydrogel for 4 days at 37 °C in humid 5% CO2 atmosphere as described in detail previously [1]. The number of cells was not controlled, but the spheroids were cultivated similar time in the same conditions.

Images of HepG2 cells were taken by a confocal microscope (Leica TCS SP5, HCX APO 63x/1, 30 Corr Glycerol objective; Leica Microsystems, Wetzlar, Germany). The actin filaments were stained with Alexa Fluor™ 594 Phalloidin (Invitrogen, USA) and the cell nuclei with DAPI (Invitrogen, USA) as described earlier by [1, 2]. First, the NFC hydrogel was enzymatically digested, and 3D HepG2 cell spheroids were collected into low-binding tubes. Then, the collected 3D cell spheroids were fixed with 3% PFA, and permeabilized with 0.1% Triton-

X-100 (T-8787, Sigma-Aldrich). Lastly, the actin filaments and nuclei of 3D cell spheroids were stained with Alexa Fluor™ 594 Phalloidin (1:100 in 1xPBS) and DAPI for 30 minutes, respectively. In the microscope, a laser DPSS with the power of 20 mW and wavelength of 561 nm, and a UV diode with the power of 50 mW and wavelength of 402 nm were used as the light source for Alexa Fluor 594 and DAPI, respectively.

2.2 Independent datasets

The nuclear staining channel of four additional volumetric datasets was used to evaluate the proposed method. These datasets were not used during the development of our system and thus we call these independent datasets. The first one, neurosphere, is a cancer spheroid composed of 52 cells imaged with a light-sheet fluorescence microscope [3]. It is publicly available at `http://opensegspim.weebly.com/download.html`. The second one is a mouse embryo imaged using confocal microscope [4, 5]. It is composed of 56 cells and is publicly available in original form at `https://figshare.com/articles/Raw_images_for_Saiz_et_al_2016/3767976/1`. The neurosphere and embryo datasets were used for evaluation in [4, 5]. These datasets as well as manually created ground truths and segmentations obtained using several software tools by [4] were downloaded from `http://www.3d-cell-annotator.org/download.html`. The third independent dataset is BBBC034 v1 dataset, which is a 3D monolayer of human induced pluripotent stem cells from the Allen Institute of Cell Sciences and is available at the Broad Bioimage Benchmark Collection (BBBC) of Broad Institute `http://https://bbbc.broadinstitute.org/BBBC034`. Ground truth labeling was downloaded from the same site. This dataset is also used in 3D segmentation tutorial at Cell Profiler [6] website `https://github.com/CellProfiler/tutorials/blob/master/3d_monolayer`. The fourth dataset, liver spheroid, is a 3D HepG2 spheroid of liver cancer cells used in first time in a earlier publication of our group [1]. This dataset is made publicly available in this study.

2.3 Manual segmentation of 12 HepG2 spheroids

The segmentation of the 12 HepG2 spheroids was performed manually using the Segment editor of 3D Slicer image computing platform [7]. Initial segmentations were done using original 1024×1024 image planes, but final checking and corrections were performed on 256×256 *input space*. The segmentation was carried out by researchers B. Omidali, T. Kaseva, U. Wilppu and E. Salli after a supervision given by the expert biologists on microscopy imaging. The boundaries of the manual segmentations were made smoother by the three-dimensional joint smoothing feature of the Segment editor. The smoothed label maps were visually checked and corrected where necessary. The number of nuclei in the ground truth spheroids varied from 76 to 225.

3 Evaluation

3.0.1 Optimization of baseline algorithms

We constructed four different watershed-based baselines for results comparisons: WS, aifWS, nlWS and blWS. The baselines were used to segment the twelve spheroids. All the baselines used the watershed method B and performed masking utilising Otsu's thresholding method [8]. aifWS, blWS and nlWS used ITK [9] implementations of various non-linear denoising techniques: gradient anisotropic diffusion filtering (aifWS) [10], bilateral filtering (blWS) [11], and patch-based denoising (nlWS) [12]. In addition, a 3D CellProfiler pipeline [13] (`https://github.com/CellProfiler/tutorials/tree/master/3d_monolayer`) was used as one of the baselines. The pipeline was tested both using the expanded data (CeP) and the original data as input (CeP non-exp).

Each algorithm had twelve different parameter settings. The parameters of each setting were tuned separately for a unique spheroid to produce a segmentation with the best PQ value. The parameters were determined using a grid search in a multidimensional parameter space. In order to estimate PQ, the ground truths were used. Consequently, the optimization process simulated a situation where an optimal method for choosing the parameters would exist.

Each algorithm, except CeP and Cep non-exp, had the same h-value range:

$$\{0.50, 0.75, 1, 00, 1.25, 1.50, 1.75, 2.00, 2.50, 3.00, 4.00, 5.00\}.$$

Due to the use of a different watershed implementation, the Cep and CeP non-exp did not use h-values but instead utilised the footprint range:

$$\{4, 8, 10, 20, 30\}.$$

.

In addition, the multidimensional parameter space with aifWS was defined as

$$\begin{aligned} \text{time step} &= \{0.015, 0.0625\} \\ \text{conductance} &= \{1, 4\} \\ \text{number of iterations} &= \{5, 10, 20\}, \end{aligned}$$

with nlWS as

$$\begin{aligned} \text{patch radius} &= \{4, 6\} \\ \text{number of sample patches} &= \{50, 100\} \\ \text{number of iterations} &= \{1\}, \end{aligned}$$

with blWS as

$$\begin{aligned}\text{domain sigma} &= \{1, 4\}\\ \text{range sigma} &= \{10, 50\}\\ \text{number of range gaussian samples} &= \{25, 100\}\end{aligned}$$

and with Cep and Cep non-exp as

$$\begin{aligned}\text{downsampling factor} &= \{1, 2\}\\ \text{median filter size} &= \{1, 3, 5\}\end{aligned}$$

For the explanation of parameters and implementations refer to the corresponding references and the API documentation of Insight Toolkit at `https://itk.org/Doxygen/html/index.html` and CellProfiler at `https://cellprofiler.org/`.

References

[1] Vappu Reijonen, Liisa K Kanninen, Eero Hippeläinen, Yan-Ru Lou, Eero Salli, Alexey Sofiev, Melina Malinen, Timo Paasonen, Marjo Yliperttula, Antti Kuronen, et al. Multicellular dosimetric chain for molecular radiotherapy exemplified with dose simulations on 3D cell spheroids. *Physica Medica*, 40:72–78, 2017.

[2] Madhushree Bhattacharya, Melina M Malinen, Patrick Lauren, Yan-Ru Lou, Saara W Kuisma, Liisa Kanninen, Martina Lille, Anne Corlu, Christiane GuGuen-Guillouzo, Olli Ikkala, et al. Nanofibrillar cellulose hydrogel promotes three-dimensional liver cell culture. *Journal of controlled release*, 164(3):291–298, 2012.

[3] L. Gole, K. H. Ong, T. Boudier, W. Yu, and S. Ahmed. OpenSegSPIM: a user-friendly segmentation tool for SPIM data. *Bioinformatics*, 32(13):2075–2077, 07 2016.

[4] F. Piccinini, T. Balassa, A. Carbonaro, A. Diosdi, T. Toth, N. Moshkov, E. A. Tasnadi, and P. Horvath. Software tools for 3D nuclei segmentation and quantitative analysis in multicellular aggregates. *Comput Struct Biotechnol J*, 18:1287–1300, 2020.

[5] E. A. Tasnadi, T. Toth, M. Kovacs, A. Diosdi, F. Pampaloni, J. Molnar, F. Piccinini, and P. Horvath. 3D-Cell-Annotator: an open-source active surface tool for single-cell segmentation in 3D microscopy images. *Bioinformatics*, 36(9):2948–2949, 05 2020.

[6] Claire McQuin, Allen Goodman, Vasiliy Chernyshev, Lee Kamentsky, Beth A. Cimini, Kyle W. Karhohs, Minh Doan, Liya Ding, Susanne M. Rafelski, Derek Thirstrup, Winfried Wiegraebe, Shantanu Singh, Tim Becker, Juan C. Caicedo, and Anne E. Carpenter. Cellprofiler 3.0: Next-generation image processing for biology. *PLOS Biology*, 16(7):1–17, 07 2018.

[7] Andriy Fedorov, Reinhard Beichel, Jayashree Kalpathy-Cramer, Julien Finet, Jean-Christophe Fillion-Robin, Sonia Pujol, Christian Bauer, Dominique Jennings, Fiona Fennessy, Milan Sonka, et al. 3D slicer as an image computing platform for the quantitative imaging network. *Magnetic resonance imaging*, 30(9):1323–1341, 2012.

[8] N. Otsu. A threshold selection method from gray level histograms. *IEEE Transactions on Systems, Man, and Cybernetics*, 9:62–66, 1979.

[9] M. McCormick, X. Liu, J. Jomier, C. Marion, and L. Ibanez. ITK: enabling reproducible research and open science. *Front Neuroinform*, 8:13, 2014.

[10] P Perona and J Malik. Scale-space and edge detection using anisotropic diffusion. *IEEE Trans. on Pattern Analysis and Machine Intelligence*, 12(7):629–639, 1990.

[11] C. Tomasi and R. Manduchi. Bilateral filtering for gray and color images. In *Sixth International Conference on Computer Vision (IEEE Cat. No.98CH36271)*, pages 839–846, 1998.

[12] A. Buades, B. Coll, and J.-M. Morel. A non-local algorithm for image denoising. In *2005 IEEE Computer Society Conference on Computer Vision and Pattern Recognition (CVPR'05)*, volume 2, pages 60–65 vol. 2, 2005.

[13] Claire McQuin, Allen Goodman, Vasiliy Chernyshev, Lee Kamentsky, Beth A Cimini, Kyle W Karhohs, Minh Doan, Liya Ding, Susanne M Rafelski, Derek Thirstrup, et al. Cellprofiler 3.0: Next-generation image processing for biology. *PLoS biology*, 16(7):e2005970, 2018.
